# Supplementary material for: Putative carboxylesterase gene identification and their expression patterns in Hyphantria cunea (Drury)
Source: PeerJ. 2021 Mar 2;9:e10919. doi: 10.7717/peerj.10919 (PMC7934681; doi:10.7717/peerj.10919)
Supplement: Supplemental Information 1 [file peerj-09-10919-s001.docx]

**Table S1. CXE genes and their accession number used in phylogenetic tree.**

| Species | Gene name | Acc. number | Species | Gene name | Acc. number |
| --- | --- | --- | --- | --- | --- |
| *S. inferens* | SinfCXE1 | AII21978.1 | *C. medinalis* | CmedCXE17 | AQY62711.1 |
| *S. inferens* | SinfCXE2 | AII21979.1 | *C. medinalis* | CmedCXE18 | AQY62712.1 |
| *S. inferens* | SinfCXE3 | AII21980.1 | *C. medinalis* | CmedCXE19 | AQY62713.1 |
| *S. inferens* | SinfCXE5 | AII21981.1 | *C. medinalis* | CmedCXE20 | AQY62714.1 |
| *S. inferens* | SinfCXE6 | AII21982.1 | *C. medinalis* | CmedCXE21 | AQY62715.1 |
| *S. inferens* | SinfCXE9 | AII21983.1 | *C. medinalis* | CmedCXE22 | AQY62716.1 |
| *S. inferens* | SinfCXE10 | AII21984.1 | *C. medinalis* | CmedCXE23 | AQY62717.1 |
| *S. inferens* | SinfCXE11 | AII21985.1 | *C. medinalis* | CmedCXE24 | AQY62718.1 |
| *S. inferens* | SinfCXE12 | AII21986.1 | *C. medinalis* | CmedCXE25 | AQY62719.1 |
| *S. inferens* | SinfCXE13 | AII21987.1 | *C. medinalis* | CmedCXE26 | AQY62720.1 |
| *S. inferens* | SinfCXE14 | AII21988.1 | *C. medinalis* | CmedCXE27 | AQY62721.1 |
| *S. inferens* | SinfCXE16 | AII21989.1 | *C. medinalis* | CmedCXE28 | AQY62722.1 |
| *S. inferens* | SinfCXE18 | AII21990.1 | *C. medinalis* | CmedCXE29 | AQY62723.1 |
| *S. inferens* | SinfCXE19 | AII21991.1 | *C. medinalis* | CmedCXE30 | AQY62724.1 |
| *S. inferens* | SinfCXE20 | AII21992.1 | *D. melanogaster* | DmelCXE6 | AAA28519.1 |
| *S. inferens* | SinfCXE26 | AII21993.1 | *B. mori* | Bmace1 | NP_001037380.1 |
| *S. inferens* | SinfCXE28 | AII21994.1 | *B. mori* | Bmace2 | NP_001108113.1 |
| *S. inferens* | SinfCXE30 | AII21995.1 | *B. mori* | Bmbe2 | NP_001124351.1 |
| *S. littoralis* | SlitCXE2 | ACV60229.1 | *B. mori* | Bmae2 | AGG20204.1 |
| *S. littoralis* | SlitCXE3 | ACV60230.1 | *B. mori* | Bmae3 | NP_001121786.1 |
| *S. littoralis* | SlitCXE4 | ACV60231.1 | *B. mori* | Bmae13 | NP_001040174.1 |
| *S. littoralis* | SlitCXE5 | ACV60232.1 | *B. mori* | Bmae19 | NP_001116501.1 |
| *S. littoralis* | SlitCXE6 | ACV60233.1 | *B. mori* | Bmae25 | NP_001121784.1 |
| *S. littoralis* | SlitCXE7 | ACV60234.1 | *B. mori* | Bmae40 | NP_001116814.1 |
| *S. littoralis* | SlitCXE8 | ACV60235.1 | *B. mori* | Bmae41 | NP_001124352.1 |
| *S. littoralis* | SlitCXE10 | ACV60237.1 | *B. mori* | Bmae45 | NP_001104822.1 |
| *S. littoralis* | SlitCXE11 | ACV60238.1 | *B. mori* | Bmae47 | NP_001091834.1 |
| *S. littoralis* | SlitCXE12 | ACV60239.1 | *B. mori* | Bmae48 | NP_001165227.1 |
| *S. littoralis* | SlitCXE13 | ACV60240.1 | *B. mori* | Bmae49 | NP_001121785.1 |
| *S. littoralis* | SlitCXE14 | ACV60241.1 | *B. mori* | Bmjhe1 | NP_001037027.1 |
| *S. littoralis* | SlitCXE15 | ACV60242.1 | *B. mori* | Bmnlg1 | XP_012548358.1 |
| *S. littoralis* | SlitCXE16 | ACV60243.1 | *T. castaneum* | TcasNLG1 | XP_015835928.1 |
| *S. littoralis* | SlitCXE17 | ACV60244.1 | *T. castaneum* | TcasNLG3 | XP_008192968.1 |
| *S. littoralis* | SlitCXE18 | ACV60245.1 | *T. castaneum* | TcasNLG4 | XP_015835573.1 |
| *S. littoralis* | SlitCXE19 | ACV60246.1 | *T. castaneum* | TcasJHE | XP_015835616.1 |
| *S. littoralis* | SlitCXE20 | ACV60247.1 | *T. castaneum* | TcasACHE1 | ADU33189.1 |
| *S. exigua* | SexiCXE | ABQ59309.1 | *T. castaneum* | TcasACHE2 | ADU33190.1 |
| *C. medinalis* | CmedCXE1 | AJN91192.1 | *T. castaneum* | TcasAE | NP_001107845.1 |
| *C. medinalis* | CmedCXE2 | AJN91193.1 | *M. brassicae* | MaraCXE | AAR26516.1 |
| *C. medinalis* | CmedCXE5 | AJN91196.1 | *A. polyphemus* | ApolPDE | AAM14415.1 |
| *C. medinalis* | CmedCXE16 | AQY62710.1 |  |  |  |
