# Supplementary material for: Putative carboxylesterase gene identification and their expression patterns in Hyphantria cunea (Drury)
Source: PeerJ. 2021 Mar 2;9:e10919. doi: 10.7717/peerj.10919 (PMC7934681; doi:10.7717/peerj.10919)
Supplement: Supplemental Information 2 [file peerj-09-10919-s002.docx]

**Table S2. Primers of *H.cunea* CXE genes used for RT-qPCR.**

| Gene name | Forward primer | Reverse primer |
| --- | --- | --- |
| *HcunCXE1* | CACCGAATATGGGATTCA | TACGCTACAACTAACAGTAG |
| *HcunCXE2* | AGAGGCAATCTTCACTAC | CTGAGATTTAAGGCTCCA |
| *HcunCXE3* | GCAAGTGAGTTTCATTCTAG | GTCGGTAGCCATATATAATAATAC |
| *HcunCXE4* | GAGGATTGTCTGTATCTCAA | AGTCGTTTTCTCCACTAC |
| *HcunCXE5* | GGTACAGAACGAGTATGG | GACACATTTCGAACCAAA |
| *HcunCXE6* | GGACAAGACAACACAAAC | CCTACAAGAGACTCAACTG |
| *HcunCXE7* | ACTGGATCAAGAACAACA | GGCGATAGGTAATGGTAG |
| *HcunCXE8* | ACGCCTGACTTTTCTTTA | ACGGATGTGTATATACTCTTC |
| *HcunCXE9* | CGTGTTACTCTATTGATAACC | GCGACTTTAGTCTTATTAAATATTG |
| *HcunCXE10* | GAGCTTGAACGTATCAGA | GGGCAAGTGTATTTGTAC |
| *EF1-a* | CAAGGCTGATGGTAAATG | ACCAGGTTTAAGGATACC |
| *GAPDH* | GCGAACGTATCTGTAGTA | CTCCTCAGTGTAGTCAAG |
