# Supplementary material for: Putative carboxylesterase gene identification and their expression patterns in Hyphantria cunea (Drury)
Source: PeerJ. 2021 Mar 2;9:e10919. doi: 10.7717/peerj.10919 (PMC7934681; doi:10.7717/peerj.10919)
Supplement: Supplemental Information 3 [file peerj-09-10919-s003.docx]

**Table S3. Primers of *H.cunea* CXE genes used for RT-PCR.**

| Gene name | Forward primer | Reverse primer |
| --- | --- | --- |
| *HcunCXE1* | ACCTCTTCCACCTCCATC | GAAACAAGCCATCCGTAG |
| *HcunCXE2* | TTAAGGCTCCAAGACCAC | TTAGTGCGGCTACTTGAT |
| *HcunCXE3* | TACGGTAAGCAAGGTGAA | AACTCAATGGCATCTGCT |
| *HcunCXE4* | CTGGATACACGGAGGAGG | TGCCCAAGGACATAAAGC |
| *HcunCXE5* | AGGCATACCTTACGCTCAG | GTTGTTCGGATCACCTCC |
| *HcunCXE6* | GGGAGAAGTGGTACAGAA | GACTACAACTACGCCATT |
| *HcunCXE7* | TCTTTATTCACGGAGGCG | CACGATAGAAGCGAGGGT |
| *HcunCXE8* | CACGCTTATTACTTCTACGA | ATGCCTCTGTGGTGTTGT |
| *HcunCXE9* | ATGCGACTGTCAACGAGA | CAGAATGGCTTTGTGGAA |
| *HcunCXE10* | AGAAACGCTATCACCCAC | TTAGCCCATTTGACTCCT |
| *EF1-a* | GAAGGCAAGGCTGATGGT | GGTGGGTTGTTCTTGGAGT |
