# Supplementary figures and images for: Putative carboxylesterase gene identification and their expression patterns in Hyphantria cunea (Drury)
Source: PeerJ. 2021 Mar 2;9:e10919. doi: 10.7717/peerj.10919 (PMC7934681; doi:10.7717/peerj.10919)

A

## E-value Distribution

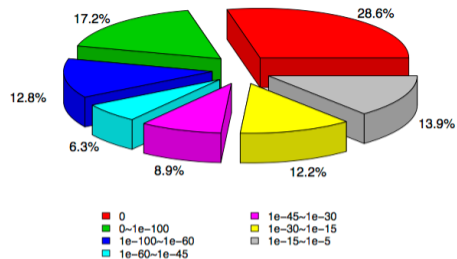

B

## Similarity Distribution

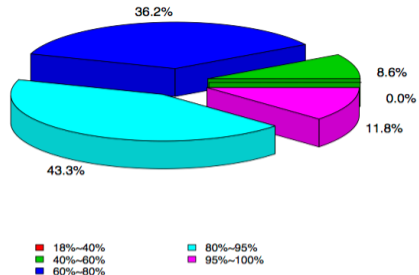

C

## Species classification

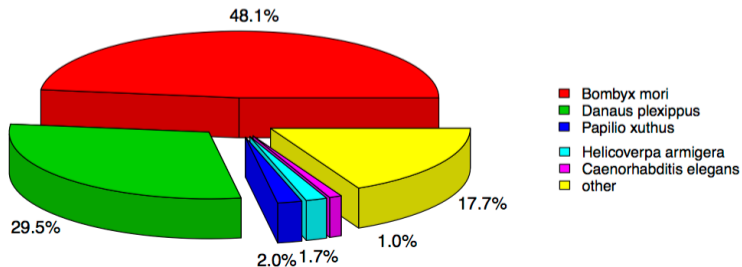

Supplement: Supplemental Information 5 — (A) E-value distribution. (B) Similarity distribution. (C) Species distribution. All unigenes that had BLASTX annotations within the NCBI nr database with a cutoff E-value of 10 −5 were analyzed. The first hit of each sequence was used for analysis. [file peerj-09-10919-s005.pdf]

Gene Function Classification (GO)

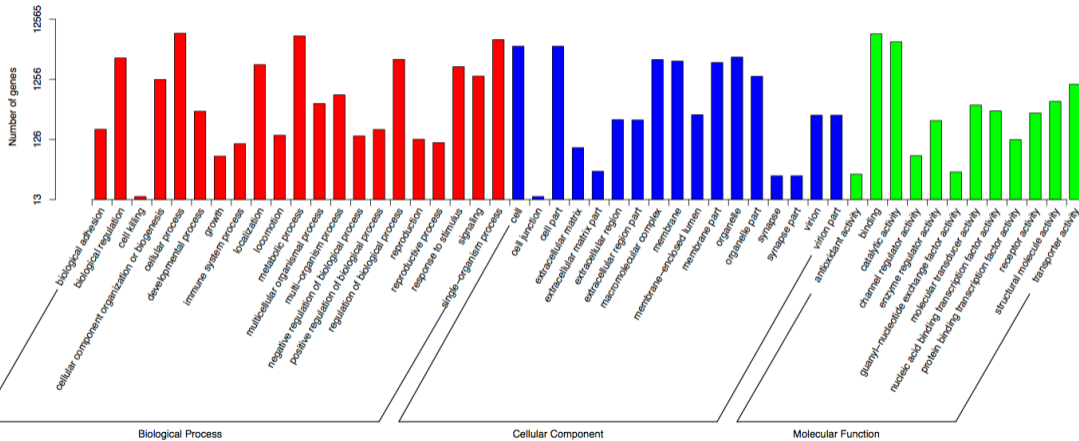

Supplement: Supplemental Information 6 — The GO classification map was done by uploading the GO ID numbers of genes for their involvement in biological processes, cellular components, and molecular functions. [file peerj-09-10919-s006.pdf]

## KEGG Classification

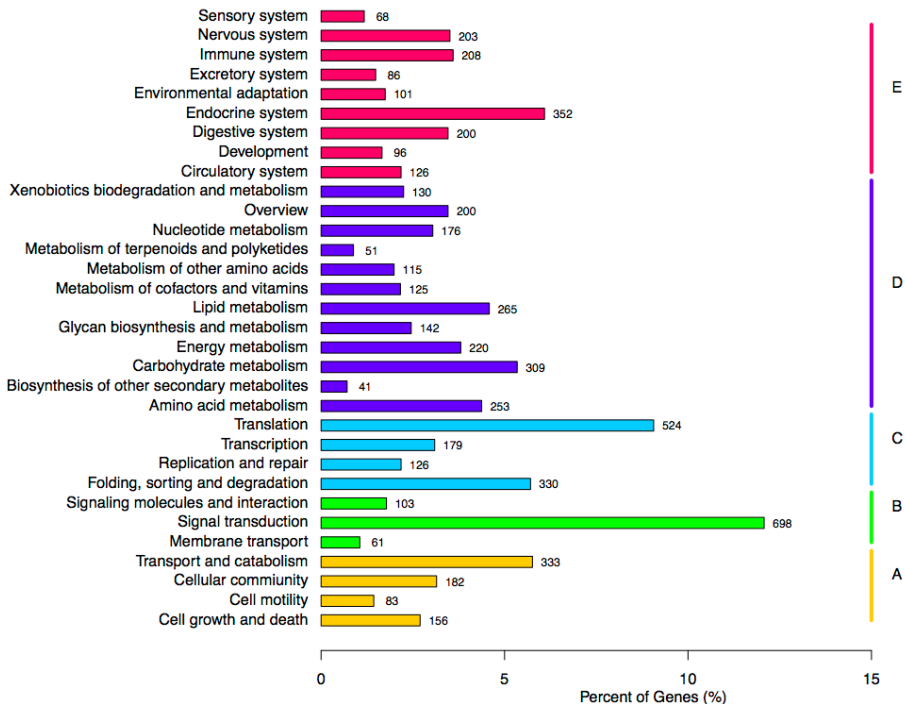

Supplement: Supplemental Information 8 — The x-axis indicates the percentage of annotated genes, and the y-axis indicates the KEGG categories. The capital letters against the colored bars indicate five main categories: (A) cellular processes, (B) environmental information processing, (C) genetic information processing, (D) metabolism, and (E) organism systems. [file peerj-09-10919-s008.pdf]

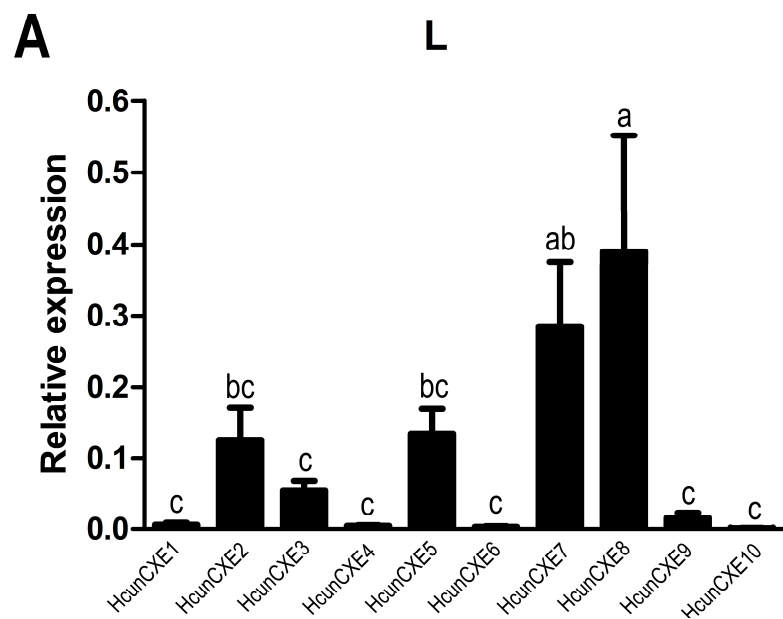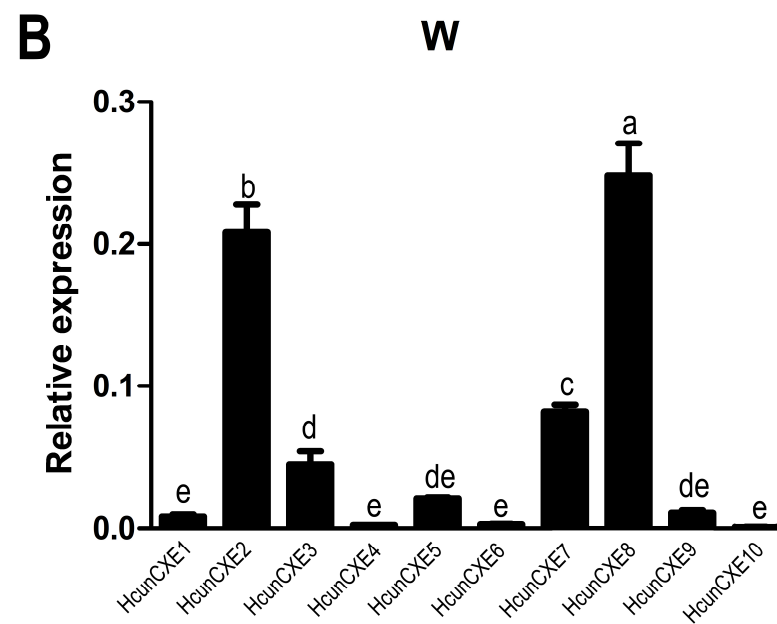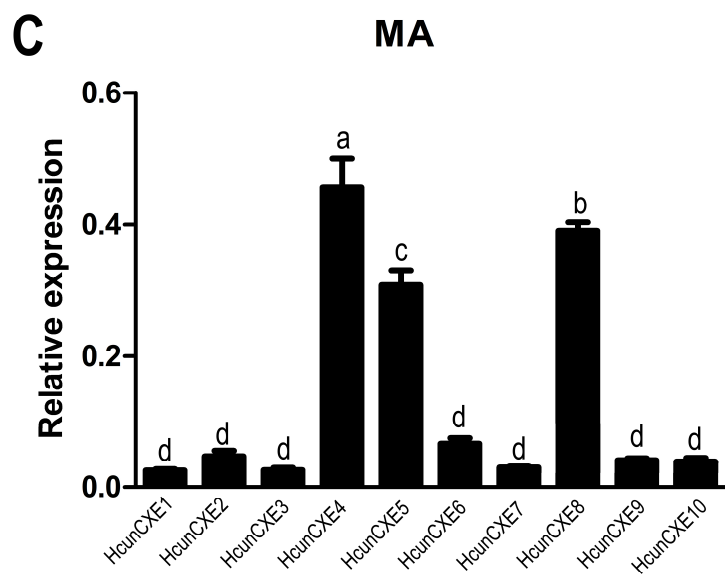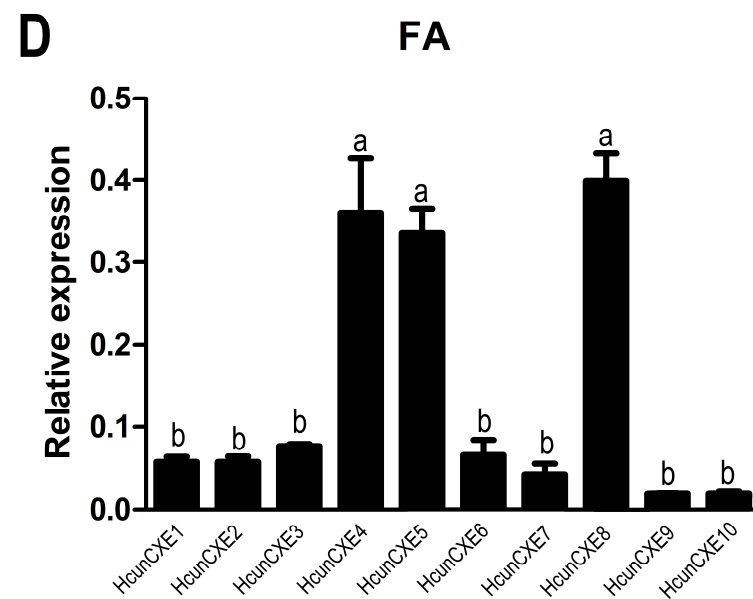

Supplement: Supplemental Information 10 — The relative mRNA levels were normalized to those of the EF1-a gene and analyzed using the Q-gene method. All values are shown as the mean ± SEM. The data were analyzed by the least significant difference (LSD) test after one-way analysis of variance (ANOVA). Different letters indicate significant differences between means (P < 0.05). [file peerj-09-10919-s010.pdf]

EF1-a

1 2 3 4 5

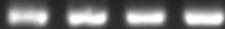

L1: Thoraxes

L2: Abdomens

L3: Pupae

L4: Larvae

L5: Negative control

Supplement: Supplemental Information 12 [file peerj-09-10919-s012.zip › Raw data supplementary material 2/Raw_data_RT-PCR EF1-a.pdf]
