# Supplementary material for: Putative carboxylesterase gene identification and their expression patterns in Hyphantria cunea (Drury)
Source: PeerJ. 2021 Mar 2;9:e10919. doi: 10.7717/peerj.10919 (PMC7934681; doi:10.7717/peerj.10919)
Supplement: Supplemental Information 7 — The letters along the x-axis represents the name of 26 groups of KOG, and the y-axis is the percentage of the number of genes annotated to the group to the total number of genes annotated. [file peerj-09-10919-s007.pdf]

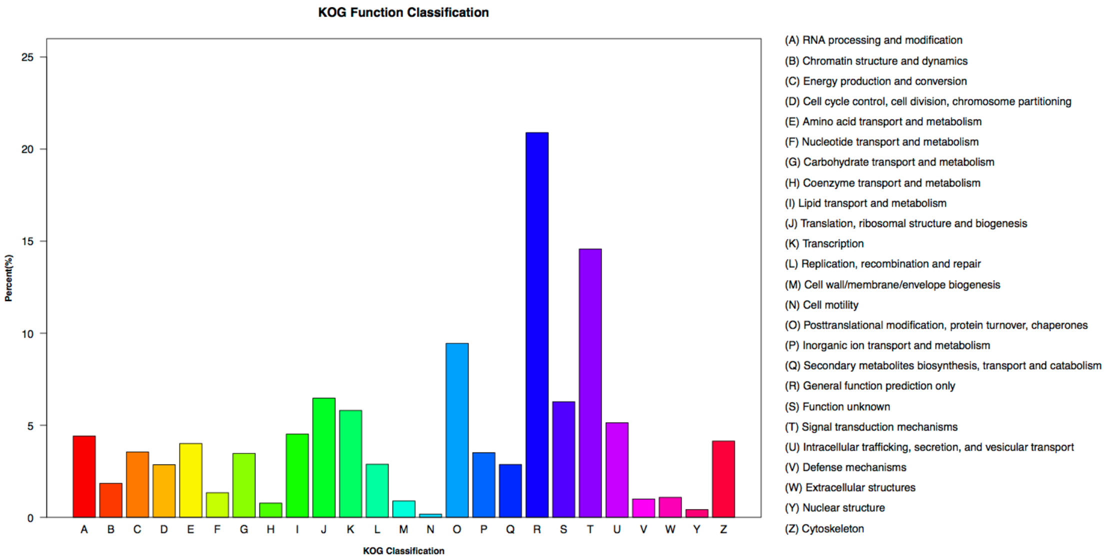
KOG Function Classification
Percent(%)
0
5
10
15
20
25
A B C D E F G H I J K L M N O P Q R S T U V W Y Z
KOG Classification
(A) RNA processing and modification
(B) Chromatin structure and dynamics
(C) Energy production and conversion
(D) Cell cycle control, cell division, chromosome partitioning
(E) Amino acid transport and metabolism
(F) Nucleotide transport and metabolism
(G) Carbohydrate transport and metabolism
(H) Coenzyme transport and metabolism
(I) Lipid transport and metabolism
(J) Translation, ribosomal structure and biogenesis
(K) Transcription
(L) Replication, recombination and repair
(M) Cell wall/membrane/envelope biogenesis
(N) Cell motility
(O) Posttranslational modification, protein turnover, chaperones
(P) Inorganic ion transport and metabolism
(Q) Secondary metabolites biosynthesis, transport and catabolism
(R) General function prediction only
(S) Function unknown
(T) Signal transduction mechanisms
(U) Intracellular trafficking, secretion, and vesicular transport
(V) Defense mechanisms
(W) Extracellular structures
(Y) Nuclear structure
(Z) Cytoskeleton
