# Supplementary material for: Putative carboxylesterase gene identification and their expression patterns in Hyphantria cunea (Drury)
Source: PeerJ. 2021 Mar 2;9:e10919. doi: 10.7717/peerj.10919 (PMC7934681; doi:10.7717/peerj.10919)
Supplement: Supplemental Information 9 — (A) HcunCXE1 with SinfCXE18; (B) HcunCXE9 and SinfCXE1; (C) HcunCXE7 with SinfCXE13 and CmedCXE5. The percentages on the right represent the amino acid identities. [file peerj-09-10919-s009.pdf]

A

```
HcunCXE1    MSSNMKYGKKIVLFSLFVTNLVDQPAPEVQIEQGILSGKINGDGSFFEYLGIPYATTNSSLRFKAPLPPPSWQGVYKAVDEIYQCPQMNP
SinfCXE18   ---MIKFGKRIILFTLFAMNLVDQPAPEVTIEQGTLSGKISTDGSFFEYVGIPYATTNSSTRFKAPLPPPSWKGVYRAVDEIYQCPQS-S

HcunCXE1    IFGVVGNEDCLKINVYVPAKIIKSALPVMVYIHGGGFTIGNGGKILYAPDFLIKHDVILVTFNYRLGALGFLCLGIKEAPGNAGIKDQIA
SinfCXE18   FIGVVGTEDCLKINVYVPA-LVKKKLPVMVYIHGRAFLLGSGGKFLYAPDFLVKHNVILVTFNYRLGALGFLCLGIKEAPGNAGLKDQIA

HcunCXE1    ALKWIKKNIGAFGGDPNNVTIFGQSAGATSASLILVSNTTDGLFHKAIIQSGISTSSWAINRQPLWVASLIAKDLGYETKNPEEIYQILS
SinfCXE18   ALRWVKRNIAAFGGDPDNVTVFGQSAGGTSVSLILASNATQGLFKKIIVQSGSSISSWAINRQPLWVASLIAKELGYDTKDPKEIFEIFS

HcunCXE1    KTSYKSLIKAKPRKPLGMYFDTQMLHYPCVEQDIEGEEAVITDFPFNLFENIPKKNIPVIYGTTSKEGIFLLPDDTKESLAARDVKYIFA
SinfCXE18   KLPYEKLVKARPKKPLGMYFDTQLLNYPCVEQEIEGEEAVITDYPYNILSNNPK-NIPAIYGTTSREGIFLIPDDTKESLAARDARYIFA

HcunCXE1    SDLQFPSEEEAANVSQMARFYYFGDKKLSFDVHDKIADLNTELYFEIPAILESQTLVNNTKAKVYNYYFNYDGGRNILKSLVGLFGFKTE
SinfCXE18   SDLEFSSENEAARVSQMMRFYFGEKNISFEVQNIITDLNTQLYFEAPAILESELLVKNTKANVHNYYFNYAGGRNFLKFIS---GFWNE

HcunCXE1    SGAVHGDELMYLFKGLVWPFPIKKNDQAIINIMTKLWTNFAKYGEPTLDDIVPIKWEPSTKESARFLYIDDNLRMGSFPNPIAFHLWKNI
SinfCXE18   AGACHSDEILYLFKGNIWNFPISKDDQNIIDWMTKMWTNFAKYGDPTPTNDLPVRWEPSTKDDMKFLYIDQDLKMGRTPNPEANRLWKNI

HcunCXE1    YEKYRKKNKPNDFMK
SinfCXE18   YEKYRKKNKPNDF--   73.9%
```

B

```
SinfCXE1    MKRTMKWLVLLSLIAARMVQQPTQPVRTRSGLVRGTVSRNGKLFQYFGIPYATVDEINRFQAPLPPPTWTGIFEAVDENTWCPQHSGG-I
HcunCXE9    ----MKWVALVFLIAAGVVRQPSPVVRTTSGYIRGAISHNGRFIEYFGIPYATVNEGSNRFQAPLPAPTWDGILNALNENTWCPQSSVGPI

SinfCXE1    IIGEPNCLKLNIYTPTRITKPLPVMVYIHGGCFFSGTGSPFLYGGDFLAENNVIFVGINYRLSVEGFLCLGIKEAPGNAGLKDQIAALKW
HcunCXE9    VLGEPNCLKLNIYSPAQYTKLLPVMVYIHGGCFFSGTGAPYLYGGDFFPEHDVILVGISYRLSVEGFLCLGIKEAPGNAGLKDVVAALKW

SinfCXE1    IQENIEAFGGDPNDVTLFGESAGAVSTSFMMLSPAAKGLFHKSILQSGSSLAPWALQHDPIETASALVKKLGYITKDPREIYNILSNKTA
HcunCXE9    IQENIKSFGGDPNKVTLFGESAGAVATSFMIISPAAKGLFHKAILQSGSSLAPWALQHDPIKAAMSLVNKLGYSANNPKDIYNIISKKTT

SinfCXE1    KEILTTINKFEERYCLAEKNLFVPCVEKPIKGVEPVVTDYPADIIKSNNYTKVPMIIGYTDKEGIYFVSADYGTSIKNNSGIIDPMKTLQ
HcunCXE9    KELINTITHYEEKFCVAETDIFVPCVERHLEGIEPIITDYPANIILSDNYTKVPMIIGYTNHEGIYFVSADYGTSLGNNTRAIDPARVLQ
```

```
SinfCXE1    EDLHFPSELDKNCTAEAIKRHYFSSYAEDLITDMVDLYSDVHIKFPLVLEAELYTRTTAQPIYYYLFRYSGLRNMPKIVSGFQFLVNGAS
HcunCXE9    RDLQFPSENDKNCTVDKIRRHYFTPSKEEMIMDMVNLYSDLHFKFPLVLESELYSRTSDQPIYYYHFKYSGLINMPKVISGFG-LSTGAS

SinfCXE1    HADELFYLFKPHAFPLLHS-AEKTMIGRMVTMWTNFAKYTDPTPRPSPLTPFRWRPSRRQNPTALIIDAQVSTAPLWEESSVRLWNDTYN
HcunCXE9    HADELFYLFKPHSFPLPHRFLESEMIKRMLTMWTNFAKYSDPTPHRSRLLPFRWNPSKRFNPTSLVIDSQISTAPMWDEDSVRLWNDTYN

SinfCXE1    KYRRKLYGEKLFQHYTDVLADGLPTS   71.3%
HcunCXE9    KYRRKDYGFKQFQHY----------
```

C

```
SinfCXE13   -MSLRLFLFLLSIAGLVFAQAFNPTVRVAQGLLQGTWKVSTKGRSYASFQGVPYARPPIGKYRFREPQHLKPWLGVWDATRPLSACLQYEP
HcunCXE7    -MIFKLFLCLVVVGLVAAQSINPTIRIPHGILQGAWKVSTKGRSYASFEGVPYARPPIGKLRFREPQQLKPWTGVWDATKPLSACLQYNP
CmedCXE5    MAALRLFSFLLLVALAAGQGSNPVVRVAHGLLQGSWKVSTNGRTYASFQGIPYARPPVGKYRFREPQQLKPWTGIWDATKPLSACLQYDP

SinfCXE13   FVKSIIGSENCLFVNVYTPKMNAGANLPVMVFIHGGAFMYGTGGIYDASNIMDWDMVVVTINYRLGPLGFLSTGDEIAPGNNGLKDQAFA
HcunCXE7    FEKKLYGSENCLYLNIHTPKLQPGAMMPVMVFIHGGAFMYGEGSIYDAPNIMDWDMVVVTINYRLGPLGFLSTGDDVIPGNNGLKDQSFA
CmedCXE5    FDKKIVGSENCLFVNVYTPNLNAGSNLPVVVFIHGGAFMYGTSSLYGAENFMDRDVVIVTVNYRLGPLGFLSSGDEQAPGNAGLKDQAFA

SinfCXE13   LHWIKNNILMFGGNPDSITLTGCSAGGASVHYHYMSPLSRGTFARGIAYSGSALTEWTHSIKPAEKARTLASIVGCPTTTNKEMMECLKY
HcunCXE7    LHWIKNNIMMFGGNPDSITLSGASAGGASVHYHYLSPLSRGTFARGIAFSGSAFASWTHSIKPAEKTKTLASIVGCPTGSSKEILECLKY
CmedCXE5    LRWVQNNIMMFGGNPNSVTLTGCSAGGASVHYHYLSPWSKGYFARGIAFSGSAFATWTHAVKAAQKARGLASIVGCPTATTREMVDCLKY

SinfCXE13   RPAEVIVNAQIEMFDWKVH-MFTPFTPVVEAPGVREPFLQQYPYHATRSGSMMNLPFITSVTSEEGLYPAAAYQETPDILPDLEANWNQL
HcunCXE7    RPGEVIVNAQADMFDWKVN-LFTPFTPVAEAWGVREPFLQYYPYHATRSGNMMNVPLIASVTSEEGLYPAAAYQTDPTILQDLEANWDEL
CmedCXE5    RPAEVLVDAQTEMFDWKPHAYFTHFTPTAEGPTVRDPFLTQYPYIAAKTGGMHPLPLIASITSEEGLYPAAAYHRDPNALQELETRWNEL

SinfCXE13   AANIFEYNDTLPLNILRNEVAMKIKQHYLGGKPVSQETFAQLVQALSDRLFVADVGKTAQLHAAKSGQPTYVYRYAFRGATSLSNLMAHND
HcunCXE7    AANIFEFNDTLPINRRVEVAQAIKQYYLGGQPVSQDTFPQLVQALGDRLFVANVGKLAQIHAAKSGQPTYVYRYAYRGSFSLSDVISQSK
CmedCXE5    ASNIFEYNDTLPVHQRASVAAQIKQKYLNNKPVSQATFPQLVQALGDRLFGSEVGRLAQTHAQRSGQPVYVYRYSHRGSISLSNFLANNN

SinfCXE13   ANYGVSHADDVLRIFKYPGLASNNPQDVAMTEGLINMWYSFSTTGIPKLINDGPAWDPVKPGAPELHYLDILSPTSMEMNSSTDFGQRSF
HcunCXE7    VNYGVSHGDDVFSIFKFPELQVKSAEHAAMVDALINIVYSFSTSGTPKLTQGGPEWLPVRPGAAELDYLHILSPNYFEMKSSSDFGLKSF
CmedCXE5    DNYGVSHADDVIAMFKFPGLDFTTTDDKKMMNALLDMVYSFATTGVPRLSNTGPTWLPVTAGSPELNYLEISSPTKHEMKTSTDFGHKSF

SinfCXE13   WDSLGFNENENYQTYLKDEL   74.6%
HcunCXE7    WDSLGFNENENYRNSIKDEL
CmedCXE5    WDSLGFIENDRFHYSIRDEL   65.0%
```
