# Supplementary material for: Putative carboxylesterase gene identification and their expression patterns in Hyphantria cunea (Drury)
Source: PeerJ. 2021 Mar 2;9:e10919. doi: 10.7717/peerj.10919 (PMC7934681; doi:10.7717/peerj.10919)
Supplement: Supplemental Information 12 [file peerj-09-10919-s012.zip › Raw data supplementary material 2/Raw_data_RT-PCR HcunCXE1-10.pdf]

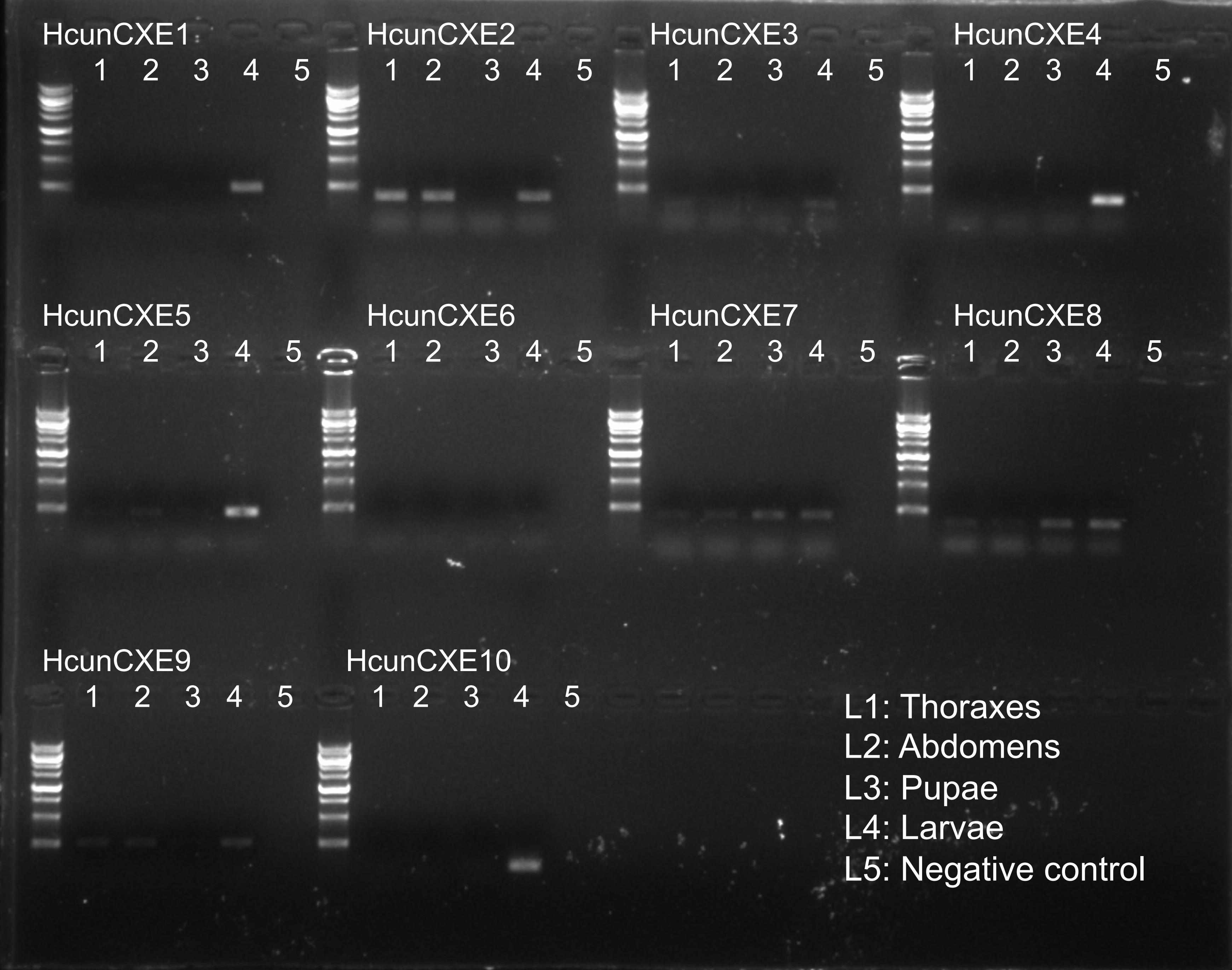
HcunCXE1
1 2 3 4 5
HcunCXE2
1 2 3 4 5
HcunCXE3
1 2 3 4 5
HcunCXE4
1 2 3 4 5
HcunCXE5
1 2 3 4 5
HcunCXE6
1 2 3 4 5
HcunCXE7
1 2 3 4 5
HcunCXE8
1 2 3 4 5
HcunCXE9
1 2 3 4 5
HcunCXE10
1 2 3 4 5
L1: Thoraxes
L2: Abdomens
L3: Pupae
L4: Larvae
L5: Negative control
